# Developing and pretesting a sexual reproductive health (SRH) school health promotion intervention (Safer Choices program) in rural South Africa: Study Protocol

**DOI:** 10.12688/wellcomeopenres.25011.1

**Published:** 2026-02-26

**Authors:** Natsayi Chimbindi, Faith Magut, Ursula Ngema, Bongimpilo Zulu, Janet Seeley, Andrew Gibbs, Abigail Harrison, Alun Davies, Maryam Shahmanesh

**Affiliations:** 1 Africa Health Research Institute, Durban, KwaZulu-Natal, South Africa; 2 University College London Institute for Global Health, London, England, UK; 3 University of KwaZulu-Natal School of Nursing and Public Health, Durban, KwaZulu-Natal, South Africa; 4 London School of Hygiene and Tropical Medicine Department of Global Health and Development, London, England, UK; 5 Gender and Health Research Unit, South African Medical Research Council, Pretoria, South Africa; 6 University of Exeter, Exeter, England, UK; 7 Brown University, Providence, Rhode Island, USA; 8 University of Oxford, Oxford, England, UK

**Keywords:** Adolescents, school learners, whole-school, HIV, sexually transmitted infections, Safer Choices, intervention

## Abstract

**Background:**

Young people remain at highest risk of HIV, sexually transmitted infections (STIs) and early pregnancy. The WHO Global School Health Initiative acknowledges the opportunity to promote effective health interventions within the sustainable infrastructure of schools. Safer Choices is a school-based multiple-component whole-school intervention effective in reducing sexual risk behaviours and preventing HIV, STIs and pregnancy among high-school learners. The overall aim of this study is to adapt and evaluate a Safer Choices program to improve uptake of sexual reproductive health (SRH) and HIV-prevention amongst 15-19-year-old learners in rural KwaZulu-Natal, South Africa.

**Methods:**

This study will use a mixed-method study design guided by the MRC-framework for development and evaluation of complex interventions. We will use the Framework for Reporting Adaptations and Modifications to Evidence-based Implementation Strategies (FRAME-IS) to document modifications to Safer Choices. The study will be conducted across six purposively selected high schools in uMkhanyakude district. The study will be divided into three work-packages (WP): WP1 to adapt and refine Safer Choices to context using participatory research methods; WP2 to pilot and assess acceptability and feasibility of the adapted intervention followed by a process evaluation to understand reach, uptake, acceptability and feasibility of intervention. We will conduct pre/post intervention surveys with 390 randomly selected learners aged 15-19 to measure exposure, satisfaction, changes in self-reported SRH and HIV-risk behaviours. WP3 will include collection of exploratory data on the effect of the intervention on improving resilience and uptake of HIV-prevention and SRH services 60-days post-intervention delivery.

**Discussion:**

This study protocol paper reports the details of our design for adaptation and evaluation of Safer Choices, with the aim of informing efforts elsewhere and scale-up of this evidence-based intervention. Integrating Safer Choices with SRH and HIV services will likely amplify its effectiveness, offering a holistic framework that addresses the multifaceted nature of HIV prevention.

## Plain Language summary

Young people in rural KwaZulu-Natal continue to face high risks of HIV, sexually transmitted infections (STIs), and early pregnancy. This study aims to adapt and evaluate an evidence-based school-based multiple-component whole-school program called Safer Choices amongst 15-19-year-old learners in rural KwaZulu-Natal, South Africa to improve uptake of sexual reproductive health (SRH) and HIV-prevention. The study will be conducted across six purposively selected high schools in uMkhanyakude district. The study will be conducted in three work-packages (WPs); WP1 to adapt and refine the Safer Choices program to fit the local context, WP2 to pilot and assess acceptability and feasibility of the Safer Choices and conduct pre/post intervention surveys with 390 randomly selected learners aged 15-19 to measure exposure, satisfaction, changes in self-reported SRH and HIV-risk behaviours. WP3 will measure effect of the intervention on improving resilience and uptake of HIV-prevention and SRH services 60-days post-intervention delivery. An adapted Safer Choices program integrated within SRH and HIV services will likely to be effective and offer a holistic framework that addresses the multifaceted nature of HIV prevention.

**List of abbreviations**
AGYW: Adolescent Girls and Young Women
AHRI: Africa Health Research Institute
ART: Antiretroviral Therapy
BREC: Biomedical Research Ethics Committee
CAB: Community Advisory Board
CT: Chlamydia Trachomatis
DREAMS: Determined Resilient Empowered AIDS-free Mentored Safe
FRAME-IS
: Framework for Reporting Adaptations and Modifications to Evidence-based Implementation Strategies
HDSS: Health and Demographic Surveillance System
HIV: Human Immunodeficiency Virus
HSV-2: Herpes Simplex Virus-2
IDI: In-depth interview
ISHP: Integrated School Health Programme
KZN: KwaZulu-Natal
LO: Life Orientation
MRC: Medical Research Council-framework
NG: Neisseria Gonorrhea
PEU: Public Engagement Unit
PEPFAR: President’s Emergency Plan for AIDS Relief
PrEP: Pre-Exposure Prophylaxis
REDCap: Research Electronic Data Capture
SGB: School Governing Board
SRH: Sexual Reproductive Health
STI: Sexually Transmitted Infections
ToC: Theory of Change
TV: Trichomonas Vaginalis
WHO: World Health Organisation
WP: Work Package

## Introduction

Despite the availability of effective HIV prevention including HIV pre-exposure prophylaxis (PrEP) and contraception, young people remain at highest risk of HIV and early and unplanned pregnancy. ^1^ In rural KwaZulu-Natal, South Africa, adolescent girls aged 15-19 bear a significant burden of HIV and have huge unmet sexual health needs. ^2, 3^ South Africa has the largest number of people living with HIV worldwide with about 7.6 million people living with HIV ^4, 5^ and KwaZulu-Natal province has the highest HIV prevalence rates in the country with an HIV prevalence of 27.8%. ^6^ HIV incidence among 15-19 year-olds was approximately 3.00/100 per 100 person years, incidence of teenage pregnancy was 6% per-annum, herpes simplex virus-2 (HSV-2) incidence was 17.3/100 person years and one-in-five 15-24 year-olds had a treatable sexually transmitted infection (STI) in uMkhanyakude district of KwaZulu-Natal as of 2019. ^2, 3, 7^ Despite the high rates of pregnancy, HIV and other STIs, only 15% of this population reported using contraception, less than 50% reported condom use during their last sexual encounter, and just 32% had tested for HIV in the past 12 months. ^2^ This highlights the existing significant gaps in adolescent sexual and reproductive health and emphasizes the need for targeted interventions to address these gaps.

The WHO's Global School Health Initiative recognises schools as a missed opportunity to deliver effective health interventions within sustainable infrastructure. ^8– 11^ In South Africa, more than 90% of school-going-age (7-19 years) young people are in school, ^12^ creating a socio-biological window of opportunity to promote healthy-behaviours and long-standing impact. ^13– 15^ Although, there is evidence of high uptake of combination HIV prevention interventions delivered through schools in uMkhanyakude district ^16^ through the PEPFAR-funded DREAMS, there still are gaps in how to scale up these interventions and lack of clear pathways to link learners with sexual reproductive health (SRH) services, more so in a context of identified cluster of health-related risks in school-going age young people. ^17– 19^ School environments provide a unique setting to build resilience and support for adolescent health, the WHO’s Health Promoting Schools (HPS) framework is a holistic, settings-based approach to promoting health and educational attainment in schools. There is evidence that well developed theoretical interventions based on the Health Promoting Schools framework can improve health outcomes for learners. ^11^

In South Africa, the Departments of Basic Education and Health are jointly implementing the Integrated School Health Programme (ISHP) to provide school health services including health education, health screening and some on-site health services to all learners in primary and secondary schools. ^20^ The aim of the ISHP is to improve children’s health, reduce health barriers to learning, and assist learners to stay in school and perform to the best of their abilities. However, the current integrated school health initiative in South Africa does not manage learners with HIV or provide sexual reproductive health (SRH) services, hence the need for adapting effective health interventions that use an eco-holistic approach such as the WHO Health Promoting Framework ^11^ that recognises the different levels of influence of health and well-being from the individual, school environment, and the wider community context and the need to act at all three levels to successfully influence health.

School-aged adolescents are an important target population for health promotion programs, particularly programs addressing sexual behaviours. In 2005, the South African government integrated the subject Life Orientation (LO), delivering HIV prevention education, into the South African school curriculum. ^21^ Although LO was successful in improving HIV-related knowledge and attitudes, marginal success was observed in reducing sexual risk behaviours and it had no impact on SRH. ^22^ Evidence suggests peer-led interventions improve knowledge, sexual-behaviour and condom-use ^14, 23, 24^ specifically, strategies that incorporate gender norms, ^13, 25– 28^ social cohesion and peer support/mentorship. ^29^ These findings suggest adapting theoretically derived multi-component whole school-promotion programs to specific settings considering the context is key to enhance acceptability and effectiveness in reducing sexual risk and improving SRH outcomes among school-going children.

Building on this evidence, we aim to adapt to context and setting, the Safer Choices program ^30^ a theory-based multicomponent educational program aimed at reducing sexual risk behaviours and increase protective behaviours in preventing HIV, sexually transmitted infections and pregnancy among high-school students to ensure that it is acceptable and feasible to deliver in this rural setting. A systematic review using the WHO Health Promoting Schools framework found positive effects of Safer Choices ^28^ but to-date Safer Choices has not been adapted for South Africa or integrated with biomedical HIV-interventions. This paper is a protocol of the study which aims to describe the process to adapt and evaluate this school health-promotion intervention to improve sexual and reproductive health and uptake of HIV-prevention amongst 15-19-year-old learners in rural KwaZulu-Natal, South Africa.

### Objectives

The overarching goal of this study is to adapt and evaluate a scalable and sustainable school health-promotion intervention (Safer Choices) to improve uptake of SRH and HIV-prevention amongst 15-19-year-old learners in rural KwaZulu-Natal.

This study has three research objectives which are: 1) to adapt and optimise the Safer Choices (a school-based program effective in reducing sexual risk-behaviours, sexually transmitted infections and pregnancy among high-school students) to include biomedical HIV-prevention; 2) to pilot the adapted intervention to assess feasibility and acceptability and 3) to provide exploratory data on the effect of intervention on sexual behaviour change and uptake of SRH or biomedical HIV-prevention, to inform future trials and scale-up.

## Methods

### Study design

This study will utilise a mixed-method study design and will be guided by the MRC-framework ^31^ for the development and evaluation of complex interventions. We will use the Framework for Reporting Adaptations and Modifications to Evidence-based Implementation Strategies (FRAME-IS) ^32^ to document modifications to implementation strategies of Safer Choices that is what is modified, the nature of the modification, the primary goal and rationale for the modification, timing of the modification, participants in the modification decision-making process, and how widespread the modification is.

### Study setting and population

The Africa Health Research Institute (AHRI) is located in Hlabisa sub-district in uMkhanyakude district, northern KwaZulu-Natal. AHRI runs a health and demographic surveillance system (HDSS) site which covers ~800km ^2^ with a population of approximately 140,000 members of 12,000 households. ^33, 34^

The uMkhanyakude district is predominantly rural with high levels of youth unemployment, significant HIV-burden, mental ill-health and teenage pregnancy in adolescents. ^2, 35, 36^ Healthcare is mainly nurse-led and provided through the public-sector fixed and mobile clinics with very little or no school-based delivery of health services except for screenings done through the Integrated School Health Teams in primary and secondary schools, including sexual and reproductive health services for older learners. ^20^

### Study population

The study will be conducted in the AHRI demographic surveillance site across six high schools purposively selected from the HDSS, to reflect a range of rural/semi-rural schools. The study area is served by the Mtubatuba and Hlabisa education circuits which are clusters of schools within a district that report to the district education office. These circuits are comprised of about 70 schools, including primary and secondary schools, predominantly located in rural areas, with a few independent and semi-private institutions. The population eligible for this study include all school-going learners in Grade 9 and 10, aged 15-19 years residing and attending school in rural KwaZulu-Natal.

### Study intervention

**Safer Choices program**

Safer Choices is a school-based multi-component intervention aimed to reduce sexual risk behaviours and increase protective behaviours in preventing HIV, sexually transmitted infections and pregnancy among high-school students. ^30^ Specifically, the Safer Choices program is designed to reduce the number of high school students engaging in unprotected sexual intercourse in two ways: by reducing the number of students who begin or have sexual intercourse during their high school years, and by increasing use of condoms and other methods of protection among students who do have sex. The program also seeks to modify several factors related to sexual risk-taking behaviour including knowledge about HIV and other STIs, attitudes about sexual behaviour and condom use, normative beliefs regarding sexual intercourse and condom use, students' beliefs in their ability to refuse sexual intercourse or unprotected sexual intercourse, use a condom, and communicate about safer sexual practices, perceived barriers to condom use, perceived risk of becoming infected with HIV or other STIs and communication with parents. The Safer Choices intervention is based on social cognitive theory, social influence theory, and models of school change. ^37^ The social cognitive theory describes how people regulate their behaviour through control and reinforcement to achieve goal-directed behaviour that can be maintained over time. ^38^ It also involves the continuous interplay between personal beliefs (e.g., self-efficacy), observable actions/behaviours (e.g., practicing a skill), and external conditions/environment (e.g., accessibility of resources), which are key principles in designing interventions and can guide a holistic approach when tackling real-world challenges.

Safer Choices consists of five main components:

1. **School Organization:** The program begins with schools establishing a Health Promotion Council, composed of teachers, students, parents, administrators, and community members. The Council has lead responsibility for organizing and planning the program activities.
2. **Curriculum:** The sequential 21-session classroom curriculum for Grade 9 and 10 (that is second and third year high school level respectively) is taught over two years (10 lessons for Grade 9 and 11 for Grade 10). It uses interactive activities to provide information about HIV and STIs; teach effective condom use, refusal skills, and decision-making skills; and promote positive attitudes and norms about refusing sex and using condoms. The curriculum includes in-class peer leaders to facilitate selected activities (e.g., leading small-group role playing) and is implemented by classroom teachers.
3. **Peer Resources and School Environment:** Each school convenes a student organization/peer team/club to reinforce the curricular messages in the broader school environment through school-wide activities, events, and services designed to alter the normative culture of school. Suggested activities include publishing articles in school media, presenting dramatic skits, or organizing speakers and assemblies.
4. **Parent Education:** Schools engage parents in the program’s goals through newsletters, child-parent discussions on sexuality topics, and parent education workshops or speakers. Newsletters provide information about the program; functional information regarding HIV/AIDS, other STIs, and pregnancy; and tips on talking with teenagers about these issues. The curriculum includes student-parent homework activities to facilitate communication regarding HIV, other STIs and pregnancy. Parents also serve on the health promotion councils and help plan other parent-related events.
5. **School-Community Linkages:** This component seeks to connect students with community resources such as local information hotlines, clinics, and testing services. Students receive homework assignments that require them to find out more about the services available in the community. These activities are designed to enhance students' familiarity with and access to support services outside school. We will use this component to deliver mobile adolescent-youth friendly SRH and HIV biomedical services through our AHRI research clinical services to optimise Safer Choices. All learners will be encouraged and supported to visit the adolescent and youth friendly sexual health services and will be provided with information on the services provided at the mobile clinic.

### Study procedure

**Figure 1.**
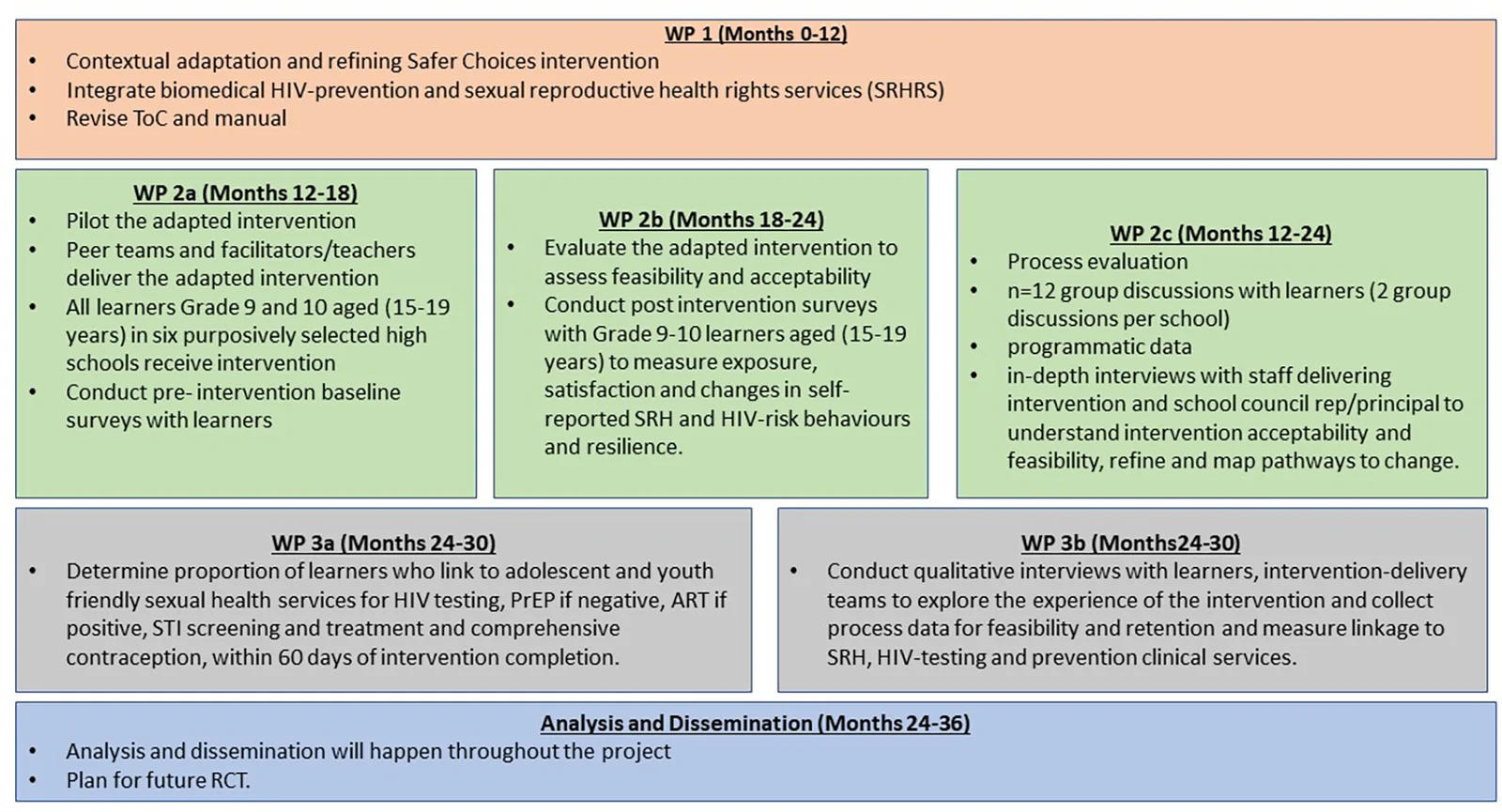
Workflow diagram of the development and evaluation of Safer Choices intervention. Figure 1 shows the different work-packages and the timelines across the study. Analysis and dissemination will happen throughout the life of the study.

**Figure 2.**
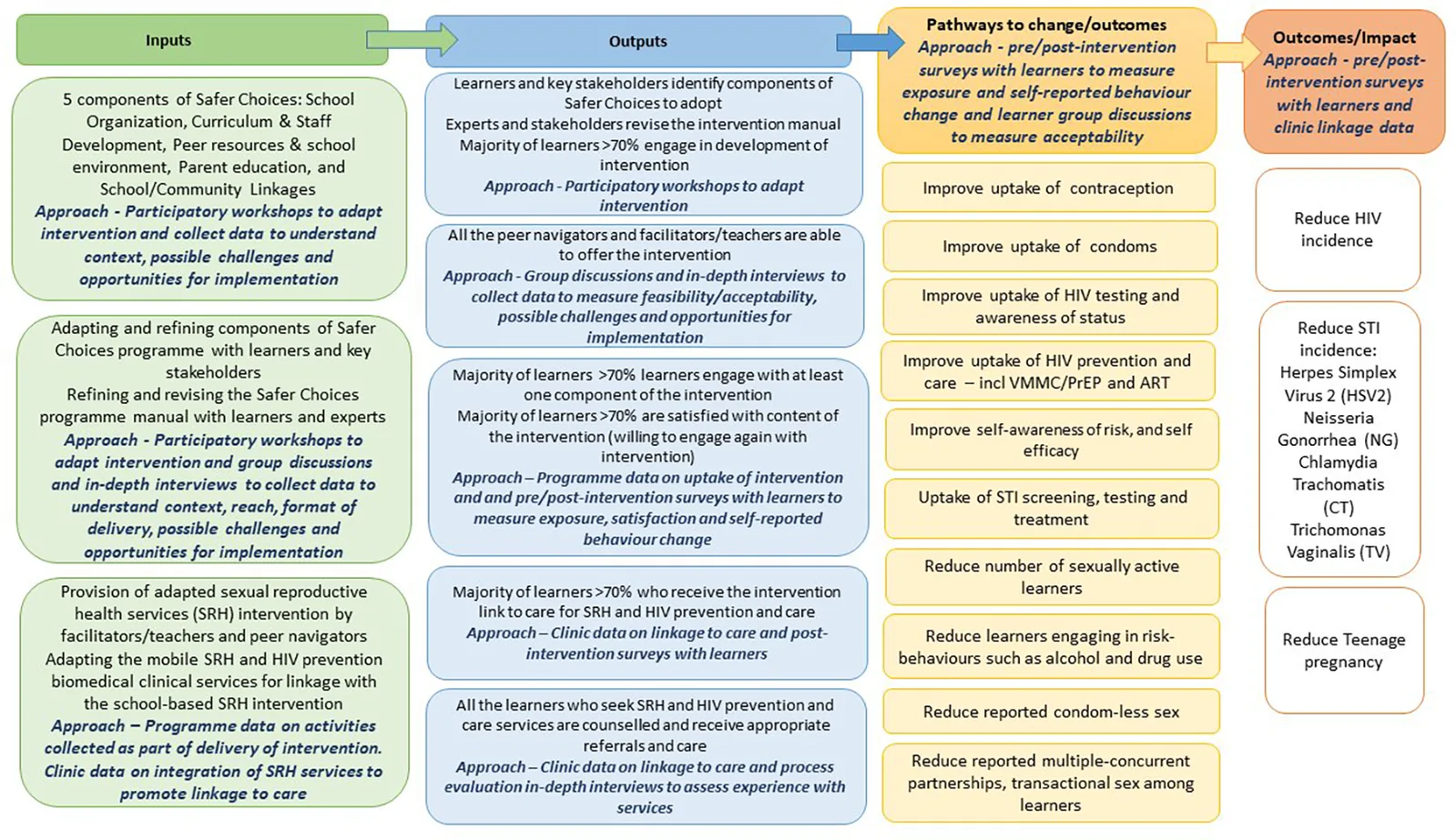
Theory of change for Safer Choices intervention. Figure 2 shows how the various study approaches and methods have been mapped to test each component in the ToC.

This study will be conducted in three multi-component work-packages (WP) over three years ( Figure 1). *In the first WP (12 months),* we will use participatory research methods to integrate biomedical HIV prevention and adapt the Safer Choices intervention to the rural context of KwaZulu-Natal. We purposively selected six high schools within the AHRI HDSS, identified by location to represent rural/semi-rural settings. The schools were selected in collaboration with the AHRI Public Engagement Unit (PEU), AHRI Community Advisory Board (CAB) and Department of Education stakeholders. We approached the Department of Education gatekeepers (the district circuit office) and informed them of the study and obtained permission to contact schools and then we approached each school to introduce the study, the study team, build relationships and get their buy-in. We will also present to parents and school governing board (SGB) meetings before the study commences. We will conduct participatory workshops, in-depth interviews, and discussions to refine the Safer Choices Theory of Change (ToC) ( Figure 2) and integrate biomedical HIV-prevention with learners, district and key-stakeholders.

We will conduct 3-day participatory workshops with up to 40 learners in Grade 9-10 aged 15-19, from two purposively selected high schools to represent rural/semi-rural schools. Additionally, we will carry out 8 in-depth interviews and discussions with education staff, including principals, teachers, and school governing body members to understand the school environment and get buy-in for intervention delivery. We will conduct two group discussions with community members, specifically parents of students from communities served by two of the schools, and another group discussion with peer navigators (lay youth leaders supporting peer-led intervention delivery in communities including schools), social scientists, and communication/media experts to further refine and revise the Safer Choices program through expert discussions. The workshop discussions with learners and community members aim to understand the context where the intervention will be delivered, identify facilitators and barriers for reach, understand content and format of delivery of each of the components of Safer Choices and integration with biomedical services. Participants will be approached by research assistants at school, home or a place of their choice and workshops or discussions and interviews will be held in community halls, classrooms, or convenient and appropriate places for all participants.

*In the second WP,* we will pilot and assess the accessibility and feasibility of the adapted intervention using three approaches.

*Approach 1 (6 months Intervention delivery)*

We will establish intervention delivery teams comprising of teachers and intervention support teams comprising of peer navigators, peer led teams (these are learners who are selected by their peers and teachers to champion and lead Safer Choices activities in their class and school), and school councils. We will train the intervention-delivery teams to deliver the intervention to all learners in Grade 9-10 across six selected high schools. Prior to intervention roll-out, a random sample of n = 390 learners will be selected for pre and post intervention surveys to measure exposure and changes in self-reported SRH and HIV-risk behaviors.

*Approach 2 (6 months Process Evaluation)*

We will conduct a mixed-method process evaluation that triangulates data to understand reach, uptake, acceptability and feasibility of intervention. The process evaluation aims to uncover what worked (and did not work), for whom, how, why and in what circumstances during the process of implementing the intervention in practice. ^39^ The Safer Choices program is built on a multicomponent framework designed to drive change at the learner, school, and community levels. The theory underpinning the Safer Choices program is based on the Social Cognitive Theory, which suggests that behaviour is influenced by the interplay of personal, environmental, and behavioral factors. ^37^ A quantitative process evaluation component will quantify the uptake of the intervention, and a qualitative component will explicitly explore “what works for who” from the range of actors involved including the learners, delivery staff and school council/governing board/principals.

Post-intervention surveys involve a follow-up of learners interviewed prior to intervention delivery to assess exposure, acceptability and satisfaction with intervention as well as reported change in self-reported SRH and risk behaviours. For the qualitative component, we will purposively select learners from various schools to capture diversity in school location (rural/semi-rural), age, grade, sex, and level of exposure to the intervention. Teachers and principals will be selected based on exploring different extents the schools are able to successfully implement the program, for example, schools which appear to have a successful implementation vs schools which have challenges, with interviews conducted at the start, middle and end of implementation.

We will conduct qualitative interviews to understand intervention acceptability and feasibility and pathways to change at different levels.

(1) Twelve group discussions will be conducted with learners, with two groups per school, each consisting of 8-10 students. Discussions will explore group dynamics and reactions to the intervention among those exposed and those not exposed.
(2) In-depth interviews (IDIs) will be conducted with staff delivering the intervention to understand what went well and what went less well with: three peer navigators supporting delivery in all schools, twelve staff directly delivering the intervention selected from all the six schools to include teachers delivering Safer Choices integrated with LO lessons, and peer led team members selected by the teachers and learners to support the curriculum-based and schoolwide activities. Interviews will also be conducted with at least one member of the school council/governing board/principal at each school to assess the feasibility and acceptability of Safer Choices at school organizational level and to what extent it ensures Safer Choices becomes schoolwide.
(3) IDIs will be conducted with 20 learners who will link to our mobile adolescent youth friendly services provided by AHRI clinical research (component 5 Safer Choices) to understand their experiences and satisfaction with the different components of Safer Choices including the process of being referred to and linked into the clinical services. Further, interviews will be conducted with staff providing clinical services at the mobile youth friendly services to understand feasibility and experiences delivering the biomedical provision component of the intervention.

We will collect and review programmatic data capturing activities, time spent, and resources used to triangulate with qualitative data. Structured observations of training of the intervention delivery team in each school and structured peer-observations of the intervention delivery in schools will be included as a pedagogical tool to improve delivery, but also to assess fidelity of the intervention delivery.

*Approach 3 (6 months Pre and post surveys)*

For the pre and post intervention surveys, a total of 390 learners (~65 per school) aged 15-19 in Grade 9-10 will be randomly selected from six schools. Trained research assistants will approach them at home, school or a place of their choice. They will obtain consent/assent and administer a tablet-based survey to measure exposure, satisfaction, and self-filled sections to measure changes in self-reported SRH and HIV-risk behaviors and resilience.

The surveys include items assessing demographic characteristics, sexuality-related psychosocial factors, sexual behaviours, and program exposure. The psychosocial scales include HIV knowledge, knowledge of STIs and contraception, self-efficacy in refusing sex, self-efficacy in using condoms, self-efficacy in communicating with partners, HIV risk behaviours, and other STI risk perceptions.

*In the third WP (6 months),* we will use exploratory data to determine the effect of intervention on improving resilience and uptake of HIV-prevention and SRH services. Specifically, we will determine proportion of learners who link to adolescent and youth friendly sexual health services for HIV-testing, PrEP if negative, ART if positive, STI screening and treatment and comprehensive contraception within 60 days of intervention completion. Adolescent and youth friendly sexual health services are offered through mobile research clinics in the AHRI HDSS.

### Approach and methods to be used

Table 1 shows a summary of the approaches and methods in the study to address the specific objectives.

**Table 1.** Summary of approaches and methods by specific objectives.

Specific objective	Approach and methods
1: Adapt and optimise the Safer Choices	Participatory workshops. Workshop discussions to understand the context where intervention will be delivered, identify facilitators and barriers for reach, adapt content and format of delivery of the five components of Safer Choices.
2: Pilot the adapted intervention	Peer led team hold school-wide activities and will be part of the school health promotion council. School councils identify opportunities for peer-delivery of the curriculum and support parent education. Mixed method process evaluation. Pre and post-intervention surveys - tablet-based survey to measure exposure, satisfaction, and self-filled sections to measure changes in self-reported SRH and HIV-risk behaviours and resilience.
3: Effect of intervention on sexual behaviour	Clinic data and conduct qualitative interviews with learners, intervention-delivery teams to explore the experience of the intervention and measure linkage to SRH, HIV-testing and prevention clinical services.

### Data collection

Data collection will be conducted at school, at home or in the community depending on arrangements with gatekeepers and where the participant feels comfortable. Data will be collected electronically on electronic tablets using the REDCap software ^40^ with self-filled sections for SRH and sensitive information to maintain privacy and obtain confidential information such as sexual risk-taking behaviours more accurately. All qualitative data is collected in the local language *isiZulu* using an interviewer guide and the interview/discussion is audio-recorded, and transcribed and translated into English.

### Study outcomes

The main outcome of this study is indicator of behaviour change measured as changes in self-reported SRH and HIV-risk behaviours among learners. We will measure behavioural outcomes including: condomless sex at last sex, sexual debut and number of sexual partners, knowledge of HIV status and uptake of contraception, HIV testing and other prevention methods such as PrEP.

Secondary outcomes of the study include linkage to sexual reproductive health services (condoms, contraception, STI screening and treatment) and biomedical HIV prevention (HIV testing, PrEP and ART). We will measure this outcome as the proportion of learners in Grade 9-10, aged 15-19 years exposed to the intervention who linked into mobile adolescent and youth friendly sexual health services within 60 days of intervention delivery.

Process evaluation outcomes include acceptability, reach and feasibility of the different intervention components. *Acceptability* is defined as more than 70% of learners participating in at least one component of the intervention and satisfied with intervention and or willing to engage with intervention again, and *feasibility* defined as intervention delivery in at least 5 out of 6 schools, and *reach* measured as learners attending >70% of sessions.

### Theory of change

It is envisioned that the Safer Choices intervention will reduce HIV incidence, STI incidence and teenage pregnancy among young people aged 15-19 years in the long run. Theory of Change ( Figure 2) shows how this would be achievable through provision of the adapted Safer Choices intervention by teachers/facilitators/peer led and peer navigators and integrating the SRH and HIV biomedical clinical services for linkage.

### Sample size

Based on formative work, we expect each school to have around 60-70 Grade 9-10 learners aged 15-19 years. We will use a before-after comparison in the six schools to measure the effect of the intervention on a range of SRH outcomes. In the baseline survey, based on data from our previous studies in the area, we estimate that 40% of learners will report testing for HIV in the past 6 months, 30% being sexually active, and 5% to be on either ART or PrEP. With 390 in total (65 per school) and allowing for 10% refusal/loss to follow-up, we have ≥90% power to detect an increase from 40% to 51% in the proportion testing for HIV in the past 6 months, a decrease from 30% to 21% in the proportion who are sexually active, and an increase from 5% to 10% in the proportion on PrEP or ART.

### Blinding

All participants at the selected schools will be unblinded to the intervention assignment as this is a pilot study. Further, since we are interested in the acceptability of the intervention, we cannot explore this if participants are unaware of their allocation. However, to minimise observer bias, research assistants will be blinded as to whether the participant engaged with the intervention, and with which components.

### Statistical analysis

Descriptive analysis will be used to summarise the baseline characteristics of the participants and the prevalence of the outcomes of interest at the pre and post intervention period. We will use a before-after comparison in the six schools to evaluate the impact of the intervention on a range of SRH outcomes. We will use multilevel models to determine the intervention effect, specifically random effects logistic regression while adjusting for the correlation between students within schools, as well as the correlation within students due to repeated measurements over time and potential confounders. All data will be analysed using STATA 16. ^41^

### Qualitative analysis

We will use thematic content analysis for both inductive and deductive themes from qualitative data. We will develop a qualitative data management and analysis plan that maps to social cognitive theory and the ToC and combine this deductive approach with inductive content analysis, drawing additional themes from the data. We will iteratively code the data and share with research team for reliability checking, inter-researcher agreement and cross-checking analysis processes. Constant comparison will be used as the coding and analysis is done and all analysis will be managed using Nvivo12 software. ^42^

### Data management

All data collected quantitatively will be managed using STATA version 16. The data from the client survey captured on REDCap dashboard will be exported into STATA, cleaned and analysed. Automatic checks for invalid values, internal inconsistency, implausible responses and additional data validation checks of the quantitative data will be run after data collection. Data from REDCap will be uploaded to a MySQL database server within a secure server at AHRI. Qualitative data will be stored in the form of Word files or in Excel both of which will be uploaded into Nvivo qualitative data management and analysis programme. All data will be anonymised at the time of transcription with names of participants replaced by a unique participant identifier. Any names of other persons mentioned during interviews/discussions will be recorded in the text with a single initial. Names of schools/areas will be replaced with the general location (district or ward) or pseudonym. We will ensure anonymity and confidentiality at all levels of the research process, and none of our reports, presentations or articles will contain study schools or participants identifying information.

### Ethics

The study has been presented to the AHRI Public engagement Unit and the AHRI community advisory board (CAB). Ethical approval has been obtained from the University of KwaZulu-Natal Biomedical Research Ethics Committee (BREC/00005024/2022). All staff (including those delivering the intervention) are provided with training on research ethics such as confidentiality, voluntary participation and good clinical practices. All data will be anonymised, with each participant assigned a unique identifier to be recorded on all questionnaires. Study participants will not have their names used during any stage of data collection except in consent forms. Any personally identifiable information (including names, identity numbers, address, geolocation data, telephone numbers) will be stored in a separate data enclave in the database with restricted access and identified by separate identifier.

Notification letters will be sent out to parents/guardians of all learners in Grade 9 and 10 to inform them of the Safer Choices program at school and provide an opt out option for their child if they are not interested. We have received parental waiver from the Biomedical Research Ethics Committee of the University of KwaZulu-Natal and permission from the AHRI Community Advisory Board for this study and we will continually engage with the relevant gatekeepers at the department of education and schools’ level to ensure that the rights of the learners and minors are protected. All participants above 18 years will be asked for an informed written consent to participate and those below 18 years will provide a written informed assent including to be audio recorded in participatory workshops, group discussions, and in-depth interviews. Participants will be provided with adequate information about the study, and they will be allowed to ask questions for clarification and will only be enrolled once they have the full understanding of the study procedures.

## Discussion

Adolescents in South Africa face huge, interlinked epidemics of poor SRH and high HIV-incidence in a context of effective biomedical tools that can eliminate HIV and reduce unwanted pregnancies. Therefore, improving adolescent sexual health in South Africa is a public health priority. Using a mixed-method design, and participatory approaches we will adapt and evaluate Safer Choices to this rural setting to enhance acceptability and feasibility of the intervention. We have actively and meaningfully engaged with key stakeholders at the department of education, school level and schools governing boards and community advisory boards as part of co-adapting the intervention and getting buy-in for the Safer Choice program to ensure the content and delivery is culturally acceptable.

We will continue to engage learners, parents/guardians and stakeholders throughout the study to ensure that Safer Choices is acceptable and reduces risks among young people and improves uptake of SRH and HIV prevention services.

Although there is availability of effective and free HIV and SRH services in most communities and through the public health system, pregnancy and HIV rates remain high among young people indicating the need to explore other platforms of delivery of HIV and SRH services and interventions. This study provides an opportunity to adapt and deliver timely interventions that promote the well-being of learners by integrating biomedical HIV-prevention into an effective whole-school health-promoting intervention Safer Choices to improve SRH and uptake of HIV-prevention amongst 15-19-year-old learners in rural KwaZulu-Natal. We will conduct a process evaluation along intervention delivery to identify experience of Safer Choices, barriers and facilitators of the intervention, test assumptions set out in the ToC and understand how these complement with the social cognitive theory, as well identify mechanisms/pathways of change, and identify social norms, attitudes, and behavioral capability in those delivering and receiving the intervention.

The Safer Choices intervention offers substantial policy benefits by providing a comprehensive, evidence-based approach to sexual health education that addresses both behavioral and, when integrated, biomedical components of HIV prevention. Implementing Safer Choices as a policy can equip young people with the knowledge and skills to make informed decisions, fostering healthier behaviors and reducing incidence rates of HIV, STIs and teenage pregnancies among adolescents. Integrating Safer Choices with biomedical HIV interventions, such as PrEP and HIV testing, can amplify its effectiveness, offering a holistic framework that addresses the multifaceted nature of HIV prevention.

## Ethics approval and consent to participate

Ethical approval has been obtained from the University of KwaZulu-Natal Biomedical Research Ethics Committee (BREC/00005024/2022). All staff were provided with training in research ethics including confidentiality, voluntary participation and good clinical practice. Written informed consent will be obtained from all participants aged 18+ years and for those aged below 18 years we will obtain written informed assent to participate and be audio recorded. We obtained parental waiver from the University of KwaZulu-Natal Biomedical Research Ethics Committee. The risk of harm is anticipated to be low. This research will be conducted in accordance with the Declaration of Helsinki.

## Consent for publication

Not applicable.

## Data Availability

This is a protocol study paper therefore no underlying data is associated with this paper. The derived dataset for the study and accompanying data dictionary are publicly available through the Africa Health Research Institute (AHRI) Data Repository https://doi.org/10.23664/AHRI.SAFERCHOICES.PRE.FOLLOWUP ^43^ The dataset within this repository meets open data standards, including the assignment of open licenses (CC-BY 4.0), provision of DOIs, no login requirements for access, and a commitment to long-term data preservation. The raw datasets used in the study contain sensitive identifiers and cannot be shared publicly but are available through restricted-access requests; please email RDMServiceDesk@ahri.org. Access to restricted datasets may be granted after publication, subject to approval of the proposed analyses by the Principal Investigator and the completion of a data access agreement. To protect participant confidentiality, only the derived, de-identified dataset will be shared openly. The study data collection tools and consent forms are shared as extended data. Extended data has been provided with the associated DOI https://doi.org/10.23664/ahri.saferchoices.documents ^44^ This project contains the following extended data: https://doi.org/10.23664/ahri.saferchoices.documents ^44^
1. Supplementary Figure 1. Study flow diagram 2. Supplementary Figure 2. Theory of Change 3. Supplementary Material 1. docx (Safer Choices Learner pre post survey study questionnaire) 4. Supplementary Material 2. docx (Safer Choices Learner information and consent form) Data are available under the terms of the Creative Commons Attribution 4.0 International license (CC-BY 4.0).
