# Supplementary material for: Developing and pretesting a sexual reproductive health (SRH) school health promotion intervention (Safer Choices program) in rural South Africa: Study Protocol
Source: Wellcome Open Res. 2026 Jul 17;11:153. Originally published 2026 Feb 26. [Version 2] doi: 10.12688/wellcomeopenres.25011.2 (PMC13560622; doi:10.12688/wellcomeopenres.25011.2)
Supplement: Peer review history

# Reviewer report: Reviewer response for version 2

**DOI:** 10.21956/wellcomeopenres.29984.r161330

**Published:** 2026-08-28

**Authors:** Kouamé Mathias N’Dri

**Affiliations:** 1 Institut Pasteur de Côte d'Ivoire, Abidjan, Cote d'Ivoire

**FINAL SECOND REVIEW REPORT**

**Overall rating**

 I thank the authors for the revisions made to the manuscript.

 After reviewing the revised version, I note that **the main concerns raised in my initial report have been satisfactorily addressed **. In particular, the authors have added research hypotheses, clarified the operational definitions of the main variables and outcomes, expanded the section on bias and mitigation strategies, and significantly improved the statistical analysis plan. They have also included a discussion on the generalizability of the results and a dissemination plan.

 These modifications significantly improve the clarity, methodological rigor and reproducibility of the protocol.

**Evaluation of comments made during the first review**

**1. Lack of explicit research hypotheses**

**Initial comment:**

 The objectives were well formulated, but no explicit research hypothesis was presented.

**Review assessment:**

 The authors have now formulated hypotheses corresponding to the main objectives of the study, including those concerning the adaptation and optimization of the intervention, its feasibility and acceptability, and its association with the use of sexual and reproductive health services and biomedical HIV prevention services.

**Status: Comment resolved.**

**2. Insufficient definition of variables**

**Initial comment:**

 The primary and secondary variables were not sufficiently defined at the operational level.

**Evaluation of the revision:**

 The authors have significantly improved this aspect. The main variables and outcomes are now defined operationally, specifying what is measured, how the measurement is carried out, the reference period, and the data source. This information is presented in the table dedicated to variables and outcomes.

**Status: Comment resolved.**

**3. Inadequate management of biases**

**Initial comment:**

 I had requested the addition of a section dedicated to biases and control strategies, particularly concerning:

- social desirability bias ;
- selection bias ;
- the risk of contamination between schools.

**Review assessment:**

 The authors have added a specific section devoted to potential sources of bias and mitigation strategies.

 Social desirability bias is taken into account in particular by the use of self-administered questionnaires, which is particularly relevant in a study on the sexual behavior of adolescents.

 The authors also discuss the risks associated with selection and loss of follow-up, as well as the risk of contamination between schools.

**Status: Comment resolved.**

**4. Incomplete statistical analysis plan**

**Initial comment:**

 I had asked for clarification:

- the variables included in the models;
- the adjustment strategy;
- managing missing data;
- the significance threshold.

**Review evaluation:**

 The statistical analysis plan has been substantially developed.

 The authors now specify the statistical models according to the type of variable, the adjustment variables, the consideration of the grouping of students at the school level and repeated measures, as well as the strategy for managing missing data.

 They also specify the conditions for multiple imputation, the assumption regarding missing data, sensitivity analyses, significance threshold, and confidence intervals.

 This revision directly addresses the concern raised in my first report.

**Status: Comment resolved.**

**Minor reservation: **given that the study includes only six schools and that the analyses take into account the grouping of participants at the school level, it would be useful to explicitly state whether the calculation of the sample size also took into account intra-school correlation.

**5. External validity and generalizability**

**Initial comment:**

 The limitations regarding the generalizability of the results were not discussed.

**Review assessment:**

 The authors have now added a discussion on generalizability . In particular, they acknowledge that the results obtained with school-aged adolescents in a rural setting may not be directly generalizable to out-of-school adolescents, urban areas, or other settings with different resources and implementation capacities.

**Status: Comment resolved.**

**6. Results dissemination plan**

**Initial comment:**

 No plan for disseminating the results was presented.

**Evaluation of the revision:**

 The authors have now added a dissemination plan which includes communicating the results to participating schools and communities, stakeholders in the health and education sectors, and scientific dissemination through conferences and publications.

**Status: Comment resolved.**

**Residual comments**

 After checking the various points raised during my first evaluation, I consider that **the major comments have been properly taken into account **.

 However, I recommend two final clarifications.

**1. Sample size and grouping at the school level**

 The protocol anticipates a relatively limited number of participating schools. Since the statistical analyses take into account the grouping of students at the school level, it would be helpful to clarify whether this grouping effect was considered when calculating the sample size and statistical power.

 This level of detail would allow for a better assessment of the robustness of the planned analyses.

**2. Interpretation of before/after changes**

 The design relies on a before/after intervention comparison without a control group. Therefore, the changes observed between the two periods cannot be definitively attributed to the intervention alone.

 I therefore recommend that authors remain cautious in their use of terms such as **"effect" **, **"effectiveness" **or **"impact" **of the intervention and that they present the changes observed as exploratory results associated with exposure to the intervention.

 This caveat is particularly important to avoid an excessive causal interpretation of future results.

**Conclusion**

 Overall, **the revised version represents a substantial improvement to the protocol **.

 The six main concerns raised in my first report have been taken into account:

- Research hypotheses : **resolved **;
- definition of variables: **resolved **;
- bias management : **resolved **;
- statistical analysis plan : **resolved **;
- generalizability : **resolved **;
- Dissemination plan : **resolved **.

The few residual points identified above are mainly requests for methodological clarification and **do not call into question the overall quality or scientific relevance of the protocol **.

**Final recommendation: APPROVED WITH RESERVATIONS**

 I am therefore **in favour of indexing the manuscript **, provided that the authors provide the two methodological clarifications mentioned above, in particular concerning the consideration of grouping at the school level in the calculation of the sample size and caution in the causal interpretation of the before/after design.

Is the study design appropriate for the research question?

Partly

Is the rationale for, and objectives of, the study clearly described?

Yes

Are sufficient details of the methods provided to allow replication by others?

Partly

Are the datasets clearly presented in a useable and accessible format?

Not applicable

Reviewer Expertise:

epidemiology

I confirm that I have read this submission and believe that I have an appropriate level of expertise to confirm that it is of an acceptable scientific standard, however I have significant reservations, as outlined above.

# Reviewer report: Reviewer response for version 2

**DOI:** 10.21956/wellcomeopenres.29984.r162380

**Published:** 2026-08-28

**Authors:** Jennifer N. Githaiga

**Affiliations:** 1 University of Cape Town, Cape Town, South Africa

The study protocol addresses a highly relevant and contemporary public health issue and more so in an HIV-endemic context where adolescent girls disproportionately bear the brunt of HIV infections.  Focusing on the adaptation of an integrated sexual and reproductive health programme to ensure contextual relevance and responsiveness to the needs of the target population bears promise. The proposed use of participatory methods particularly commendable, as it is likely to enhance the acceptability, feasibility, and potential impact of the adapted intervention. Overall, the protocol is clearly described, providing a persuasive rational for the study with a coherent methodological approach (and attendant frameworks) detailed in the various work packages described.

 My main queries/points of clarification relate to the methods:

**Theoretical framework**

 The authors note that the Safer Choices intervention is based on the Social Cognitive Theory, which is accurately described. Whereas this is a relevant interpersonal theory for exploring determinants of health actions/behaviour, some additional theorising around the limitations of the theory and how the study might mitigate some of these limitations may be helpful, for instance: potential challenges in establishing which of the components (cognitive/personal, behavioural or environmental) have the most influence on actual behaviour to inform the development of a tailored intervention; the heavy focus on learning processes at the expense of biological factors that may shape behaviour, and more so for adolescents; the relatively emphasis on cognitions and observations  and less on the role of emotions as drivers of behaviour (and behaviour change). 

 Given the central role of behaviour change in the proposed intervention, perhaps the authors might consider integrating a second theory (e.g. the Health Belief Model or Theory of Planned Behaviour) as an additional lens in designing and evaluating the intervention. 

**Work Package 1:**

 This section, which focuses on participatory research methods, could be further strengthened: 

 Reframing/anchoring the section on three key components of a participatory approach, namely (i) co-creation (ii) adolescents as lived experience experts of their own sexual and reproductive health experiences, and (iii) utilisation of a small pilot for iterative refinement and contextual validation. In its current form, these components are alluded to but not strongly foregrounded. 

 Acknowledging and briefly addressing around (i) power dynamics in stakeholder relationships and potential strategies to reduce power differentials (ii) social desirability, which may negatively impact co-creation of the intervention.

 Key informant interviews (as different from regular in-depth interviews) may be more valuable as a method of data collection among expert stakeholders.

 The parent education component includes communication channels such as newsletters and student-parent homework activities, which may disadvantage parents who are unable to read. Some consideration ought to be given to more inclusive strategies and more so for the rural/semi-rural population with whom the proposed intervention will be co-developed and rolled out.

**Work Package 2:**

 Approach 2 reads like a realist evaluation. If so, perhaps is should be framed as a realist process evaluation, which will add to the novelty of the study.

 More detail should be provided about the 12 (focus?) group discussions including heterogeneity versus homogeneity considerations and rationale for group selection.

Is the study design appropriate for the research question?

Yes

Is the rationale for, and objectives of, the study clearly described?

Yes

Are sufficient details of the methods provided to allow replication by others?

Partly

Are the datasets clearly presented in a useable and accessible format?

Not applicable

Reviewer Expertise:

I am a social and behavioural public health scientist and qualitative researcher.

I confirm that I have read this submission and believe that I have an appropriate level of expertise to confirm that it is of an acceptable scientific standard, however I have significant reservations, as outlined above.

# Reviewer report: Reviewer response for version 2

**DOI:** 10.21956/wellcomeopenres.29984.r161331

**Published:** 2026-07-25

**Authors:** Tonderai Mabuto

**Affiliations:** 1 The Aurum Institute, Johannesburg, South Africa

The authors have adequately addressed the comments raised in the previous review. The revisions have strengthened the manuscript, and I have no further comments.

Is the study design appropriate for the research question?

Yes

Is the rationale for, and objectives of, the study clearly described?

Yes

Are sufficient details of the methods provided to allow replication by others?

Partly

Are the datasets clearly presented in a useable and accessible format?

Not applicable

Reviewer Expertise:

Implementation Science, Mixed Methods

I confirm that I have read this submission and believe that I have an appropriate level of expertise to confirm that it is of an acceptable scientific standard.

# Reviewer report: Reviewer response for version 1

**DOI:** 10.21956/wellcomeopenres.27554.r154331

**Published:** 2026-05-15

**Authors:** Tonderai Mabuto

**Affiliations:** 1 The Aurum Institute, Johannesburg, South Africa

The study protocol addresses an important area: tailoring interventions to context in order to improve fit, which is central to implementation success and the achievement of desired individual- and population-level outcomes. The objectives of the study are clear, and the integration across the different work packages is well articulated.

 1. **Reframing claims about the intervention**

 The objectives of the study are to adapt and evaluate the *Safer Choices* intervention. At this stage, however, claims relating to scalability and sustainability may not yet be fully justified. The authors appropriately note elsewhere that the findings will inform future trials and potential scale-up efforts. In light of this, the framing of the following statement may need reconsideration:

 “The overarching goal of this study is to adapt and evaluate a scalable and sustainable school health-promotion intervention (Safer Choices) to improve uptake of SRH and HIV-prevention amongst 15–19-year-old learners in rural KwaZulu-Natal.”

 2. **Main outcomes of the study**

 The authors state that “The main outcome of this study is indicator of behaviour change measured as changes in self-reported SRH and HIV-risk behaviours among learners.” This is somewhat unclear, as the outcome appears to align primarily with the study's third objective. In contrast, Objectives 1 and 2 focus on adaptation, optimisation, and piloting of the intervention, which are more closely linked to implementation outcomes. While behavioural outcomes are undoubtedly important, the authors may wish to clarify why implementation outcomes are not given similar prominence within the framing of the study’s main outcomes.

**3. Clarity regarding the acceptability outcome**

 The process evaluation outcome relating to acceptability is not entirely clear:

 “Acceptability is defined as more than 70% of learners participating in at least one component of the intervention and satisfied with intervention and/or willing to engage with intervention again.”

 Please clarify what is meant by “participating in at least one component of the intervention.” If this refers to the five main intervention components presented earlier in the manuscript, it is not entirely clear how Component 1 (School organisation) relates to learner participation and the acceptability outcome.

 In addition, the second part of the definition, “…satisfied with the intervention and/or willing to engage with the intervention again,” may require further clarification. Specifically, it is unclear how this aspect of acceptability will be interpreted for learners who may only have engaged with selected components of the intervention rather than the intervention as a whole.

 4. **Clarification of the feasibility outcome**

 The authors define feasibility as “intervention delivery in at least 5 out of 6 schools.” This definition may benefit from further qualification, as intervention delivery alone may not necessarily indicate feasibility if implementation fidelity is low. As such, the concept of “intervention delivery” requires further explanation. Given that this is a protocol paper, it would strengthen the manuscript if the authors could provide additional detail on how feasibility will be assessed and measured in practice, particularly as this remains an evolving area within implementation research.

 5. **Purposive sampling**

 The use of purposive sampling for the selection of schools is appropriate and well aligned with the study design. The authors also clarify that the selection process involved engagement with relevant stakeholders. Given the importance of context in intervention adaptation, the manuscript will be strengthened by providing additional information on the specific considerations that informed the selection of schools, beyond the general description of a “range of rural/semi-rural schools.” For example, were factors such as prior experience with interventions, school size, resource availability, leadership engagement, or the distinction between public and independent schools considered during the selection process?

Is the study design appropriate for the research question?

Yes

Is the rationale for, and objectives of, the study clearly described?

Yes

Are sufficient details of the methods provided to allow replication by others?

Partly

Are the datasets clearly presented in a useable and accessible format?

Not applicable

Reviewer Expertise:

Implementation Science, Mixed Methods

I confirm that I have read this submission and believe that I have an appropriate level of expertise to confirm that it is of an acceptable scientific standard, however I have significant reservations, as outlined above.

# Response

**Published:** 2026-07-04

**Authors:** Natsayi Chimbindi

**Affiliations:** Faculty, Africa Health Research Institute, Durban, KwaZulu-Natal, South Africa

**Safer Choices – Reviewer 2 comments ** The study protocol addresses an important area: tailoring interventions to context in order to improve fit, which is central to implementation success and the achievement of desired individual- and population-level outcomes. The objectives of the study are clear, and the integration across the different work packages is well articulated.

 1. **Reframing claims about the intervention**

 The objectives of the study are to adapt and evaluate the *Safer Choices* intervention. At this stage, however, claims relating to scalability and sustainability may not yet be fully justified. The authors appropriately note elsewhere that the findings will inform future trials and potential scale-up efforts. In light of this, the framing of the following statement may need reconsideration:

 “The overarching goal of this study is to adapt and evaluate a scalable and sustainable school health-promotion intervention (Safer Choices) to improve uptake of SRH and HIV-prevention amongst 15–19-year-old learners in rural KwaZulu-Natal.”

 We have revised the overarching aim to emphasise the adaptation and piloting of the intervention, with a focus on feasibility, acceptability, and potential effectiveness, rather than scalability. It now reads: “The overall aim of this study is to adapt and evaluate a school-based health promotion intervention (Safer Choices) its feasibility, acceptability, and potential effectiveness in improving sexual behaviour and uptake of sexual and reproductive health (SRH) and HIV prevention services among 15–19-year-old learners in rural KwaZulu-Natal.” (Lines 65-68, page 6).  

 2. **Main outcomes of the study**

 The authors state that “The main outcome of this study is indicator of behaviour change measured as changes in self-reported SRH and HIV-risk behaviours among learners.” This is somewhat unclear, as the outcome appears to align primarily with the study's third objective. In contrast, Objectives 1 and 2 focus on adaptation, optimisation, and piloting of the intervention, which are more closely linked to implementation outcomes. While behavioural outcomes are undoubtedly important, the authors may wish to clarify why implementation outcomes are not given similar prominence within the framing of the study’s main outcomes.

 We have revised the outcomes section to better align with the study objectives, with implementation outcomes (including feasibility, acceptability) now defined and how there are measured. We have also updated the outcomes accordingly to reflect these changes in table 2, line 337, page 14-15 **Table 2: Summary of exploratory variables** **Variables** **Definition** **Data source** **Changes in sexual behaviour ** Condomless sex Proportion of sexually active learners reporting no condom use at last sexual intercourse in the past 12 months Survey data Number of sexual partners Number of sexual partners reported in the past 12 months Survey data Transactional sex Proportion of learners who received money or gifts in exchange for sex in the past 12 months Survey data Knowledge of HIV status Proportion of learners reporting awareness of their HIV status (tested and received results) within the past 12 months Survey data Uptake of HIV testing Proportion of learners reporting HIV testing within the past 12 months Survey data Uptake of PrEP Proportion of learners reporting ever use any form of PrEP Survey data Received information on SRH, HIV, and STI services Proportion of learners reporting receipt of information on at least one SRH or HIV prevention service Survey data Uptake of SRH services Proportion of learners reporting access to at least one SRH or HIV-prevention service Survey data **Health service uptake ** Contraception use Proportion of learners reporting current use of any contraception method Survey data Pregnancy Proportion of learners reporting current pregnancy at the time of the survey Survey data Linkage to SRH services Proportion of learners who accessed any SRH service (including condoms, contraception, or STI services) Survey and clinic data Linkage to HIV testing Proportion of learners who accessed HIV testing services within 60 days post-intervention Survey and clinic data Linkage to PrEP Proportion of learners initiated on PrEP within 60 days post-intervention Survey and clinic linkage data Linkage to ART Proportion of HIV-positive learners initiating ART Survey and clinic linkage data **Process evaluation ** Feasibility Proportion of eligible learners who consent to participate in the study Proportion of enrolled learners who complete follow-up surveys post-intervention Proportion of learners exposed to and completing key learner-based components of the intervention, with feasibility defined as ≥70% completion. Survey data and qualitative data Acceptability Defined as learners’ perceived satisfaction with, and willingness to engage with, the intervention components to which they were exposed. Participation will be defined as exposure to one or more learner-based components of the intervention, including curriculum-based sessions, peer-led activities, and school/community linkage components, and excluding school-level organisational and parent-focused components. Among learners exposed to at least one learner-based component, acceptability will be assessed as the proportion reporting satisfaction with the component(s) received and willingness to engage with similar activities in the future with acceptability considered achieved if ≥70% of learners meet this criterion. Survey data and qualitative data Intervention fidelity Defined as the extent to which the intervention can be delivered as intended across schools, incorporating multiple dimensions including reach (proportion of eligible learners exposed), completeness of delivery (extent to which planned components are delivered), and adherence to the intervention protocol and quality of delivery Qualitative data  

**3. Clarity regarding the acceptability outcome**

 The process evaluation outcome relating to acceptability is not entirely clear:

 “Acceptability is defined as more than 70% of learners participating in at least one component of the intervention and satisfied with intervention and/or willing to engage with intervention again.”

 Please clarify what is meant by “participating in at least one component of the intervention.” If this refers to the five main intervention components presented earlier in the manuscript, it is not entirely clear how Component 1 (School organisation) relates to learner participation and the acceptability outcome.

 In addition, the second part of the definition, “…satisfied with the intervention and/or willing to engage with the intervention again,” may require further clarification. Specifically, it is unclear how this aspect of acceptability will be interpreted for learners who may only have engaged with selected components of the intervention rather than the intervention as a whole.

   We have clarified the definition of acceptability to distinguish between exposure and perceptions. Participation is now defined in relation to learner-based components of the intervention (e.g., curriculum sessions, peer-led activities, and linkage components), explicitly excluding school-level organisational components. We have also refined the acceptability outcome to specify how satisfaction and willingness to engage are assessed among learners based on the components to which they were exposed. These revisions have been incorporated in Table 2 line 337, page 14-15 The second part of the definition “…satisfied with the intervention and/or willing to engage with the intervention again,” is based on the affective attitude construct of the Theoretical Framework of Acceptability to assess how learners “overall” felt or experienced the intervention Safer Choices regardless of the specific component (which are assessed separately) they may have engaged with.

   4. **Clarification of the feasibility outcome**

 The authors define feasibility as “intervention delivery in at least 5 out of 6 schools.” This definition may benefit from further qualification, as intervention delivery alone may not necessarily indicate feasibility if implementation fidelity is low. As such, the concept of “intervention delivery” requires further explanation. Given that this is a protocol paper, it would strengthen the manuscript if the authors could provide additional detail on how feasibility will be assessed and measured in practice, particularly as this remains an evolving area within implementation research.

 We have revised the definition to provide a more comprehensive assessment of feasibility, incorporating multiple dimensions including intervention reach, completeness of delivery, and fidelity across schools. These revisions have been incorporated into the paper in Table 2 line 337, page 14-15.

 5. **Purposive sampling**

 The use of purposive sampling for the selection of schools is appropriate and well aligned with the study design. The authors also clarify that the selection process involved engagement with relevant stakeholders. Given the importance of context in intervention adaptation, the manuscript will be strengthened by providing additional information on the specific considerations that informed the selection of schools, beyond the general description of a “range of rural/semi-rural schools.” For example, were factors such as prior experience with interventions, school size, resource availability, leadership engagement, or the distinction between public and independent schools considered during the selection process?

 We purposively selected with our AHRI Public Engagement Unit and the district education office and included public government schools that represented location - rural/semi-rural, school size – small/big schools in terms of enrolment, schools that had previously worked with AHRI or those which would be interested in working with AHRI, and schools recommended by the Community Advisory Board (CAB) and the district education office to represent the typical district schools types. We have now clarified this on page 9, line 175-179 as follows: “We purposively selected six high schools within the AHRI HDSS, identified by location to represent location - rural/semi-rural settings, school size – small/big schools in terms of enrolment, schools that had previously worked with AHRI or those which would be interested in working with AHRI, and schools recommended by the Community Advisory Board (CAB) and the district education office to represent the typical district schools types.”

# Reviewer report: Reviewer response for version 1

**DOI:** 10.21956/wellcomeopenres.27554.r151853

**Published:** 2026-04-29

**Authors:** Kouamé Mathias N’Dri

**Affiliations:** 1 Institut Pasteur de Côte d'Ivoire, Abidjan, Cote d'Ivoire

**REPORT**

**General Assessment**

 The study is relevant from a public health perspective, particularly given the high prevalence of HIV among adolescents in sub-Saharan Africa.

 However, several aspects require clarification and improvement prior to publication.

**Strengths**

- Relevance and originality

- Topic of major interest (HIV, SRH among adolescents)
- Solid methodological framework
- Use of the MRC framework for complex interventions
- Use of the FRAME-IS framework for adaptation

**Major points to be corrected **

- Lack of explicit hypotheses: The objectives are well formulated, but no research hypotheses are explicitly stated.
- Insufficient definition of variables
- The primary and secondary variables are not clearly defined in operational terms.
- Insufficient bias management: Add a section dedicated to biases and control strategies. (social desirability bias (very critical here), selection bias, cross-school contamination bias)
- Incomplete statistical analysis plan: Although models are mentioned, the following are missing: variables included in the models, adjustment strategy, handling of missing data, significance threshold)

**Minor points for improvement**

- External validity / generalizability: Limitations of generalizability are not discussed.
- Plan for dissemination of results: Not mentioned.

**Decision: Major revisions**

 The study is relevant and well-designed, but requires significant methodological improvements to ensure scientific rigor and reproducibility.

**REPORT**

**General Assessment**

 The study is relevant from a public health perspective, particularly given the high prevalence of HIV among adolescents in sub-Saharan Africa.

 However, several aspects require clarification and improvement prior to publication.

**Strengths**

- Relevance and originality

- Topic of major interest (HIV, SRH among adolescents)
- Solid methodological framework
- Use of the MRC framework for complex interventions
- Use of the FRAME-IS framework for adaptation

**Major points to be corrected **

- Lack of explicit hypotheses: The objectives are well formulated, but no research hypotheses are explicitly stated.
- Insufficient definition of variables
- The primary and secondary variables are not clearly defined in operational terms.
- Insufficient bias management: Add a section dedicated to biases and control strategies. (social desirability bias (very critical here), selection bias, cross-school contamination bias)
- Incomplete statistical analysis plan: Although models are mentioned, the following are missing: variables included in the models, adjustment strategy, handling of missing data, significance threshold)

**Minor points for improvement**

- External validity / generalizability: Limitations of generalizability are not discussed.
- Plan for dissemination of results: Not mentioned.

**Decision: Major revisions**

 The study is relevant and well-designed, but requires significant methodological improvements to ensure scientific rigor and reproducibility.

Is the study design appropriate for the research question?

Partly

Is the rationale for, and objectives of, the study clearly described?

Yes

Are sufficient details of the methods provided to allow replication by others?

Partly

Are the datasets clearly presented in a useable and accessible format?

Not applicable

Reviewer Expertise:

NA

I confirm that I have read this submission and believe that I have an appropriate level of expertise to confirm that it is of an acceptable scientific standard, however I have significant reservations, as outlined above.

# Response

**Published:** 2026-07-04

**Authors:** Natsayi Chimbindi

**Affiliations:** Faculty, Africa Health Research Institute, Durban, KwaZulu-Natal, South Africa

**Responses to Reviewer comments** **Reviewer 1** **Major points to be corrected**

- Lack of explicit hypotheses: The objectives are well formulated, but no research hypotheses are explicitly stated.

We have revised and now include study hypotheses aligned with each objective. We have stated in line 74-78 page 7 that “We hypothesise that the adapted Safer Choices intervention can be successfully adapted and optimised to include biomedical HIV prevention services. We further hypothesise that the adapted intervention will be feasible to implement in schools and acceptable to learners. Finally, we hypothesise that the exposure to the intervention will be associated with increased uptake of SRH and biomedical HIV prevention services”.

- Insufficient definition of variables
- The primary and secondary variables are not clearly defined in operational terms.

We have revised the manuscript to explicitly define all primary and secondary variables in operational terms, including how each variable is measured, the relevant timeframes, and the corresponding data sources. These details are now presented in Table 2 on page 14 line 337

- Insufficient bias management: Add a section dedicated to biases and control strategies. (social desirability bias (very critical here), selection bias, cross-school contamination bias)

We have added a section titled “potential biases and mitigation strategies” line 420-437 page 17-18, outlining key sources of bias and control strategies. We address social desirability bias by using self-administered questionnaires to enhance privacy and reporting accuracy. We also consider selection bias, including loss to follow-up, and describe both recruitment strategies and analytical approaches such as inverse probability weighting as control strategies. Given the pre–post design, cross-school contamination is less likely to bias comparisons; however, we acknowledge potential variation in exposure across schools and describe steps taken to ensure standardised implementation.

- Incomplete statistical analysis plan: Although models are mentioned, the following are missing: variables included in the models, adjustment strategy, handling of missing data, significance threshold)

We have expanded the statistical analysis section in line 363-397 page 16 to clearly specify the variables included in the models, the adjustment strategy, and the handling of missing data. We now clearly outline that the confounding variables were selected based on prior knowledge and outline the modelling approach for each outcome. In addition, we have clarified the approach to missing data, including assumptions and planned methods, and specified the significance threshold used for statistical inference. **Minor points for improvement**

- External validity / generalizability: Limitations of generalizability are not discussed.
- Plan for dissemination of results: Not mentioned.

We have now included a discussion of the study’s generalisability (line 493-501 page 19). We highlight that findings may be context-specific to the study setting and population. We describe key factors that may influence external validity, including the school-based design of the study and the characteristics of participating learners, and discuss how these may affect the applicability of results to other settings. We have also added a section (line 502-508 page 19) outlining plans for dissemination of study findings. Findings of the study will be shared with key stakeholders, including participating schools and department of health and education, and disseminated through other ways such as peer-reviewed publications and conference presentations.  

**Decision: Major revisions**

 The study is relevant and well-designed, but requires significant methodological improvements to ensure scientific rigor and reproducibility.

 Thank you for the very useful reviews which will strengthen our manuscript, we have responded point-by-point and made updates to the manuscript to increase clarity of our hypothesis and improved on the methodology section. We hope this addresses the reviewer’s concerns.
